# Point of equilibrium

**DOI:** 10.1590/2177-6709.23.2.007-008.edt

**Published:** 2018

**Authors:** Flavia Artese

**Affiliations:** 1 Universidade do Estado do Rio de Janeiro, Departamento de Odontologia Preventiva e Comunitária (Rio de Janeiro/RJ, Brazil).

Historically, humanity alternates cycles in its relentless search for explanations in the most diverse spheres, such as arts, philosophy or science. This restlessness, which is inherent to our species, seems to be followed by a certain conflict, because of ingrained beliefs or of habits that we wish to become permanent, be it for protective reasons or for comfort, or simply to establish a point of view.

When I read for the first time the transcription of the heated debate between Case, Dewey and Cryer in 1911,^1^ ^-^ ^4^ the subjectivity of the opinions in favor or against tooth extractions for orthodontic purposes really made an impression. This could be explained by the fragility of science at that time, but the amount of “nevers” and “always” are very clear on each side of the debacle.

Along the history of our specialty, this pendulum of changes fortunately continues to swing. Since the invention of cephalometry and its adoption by Tweed as a diagnostic tool, his non-extraction treatments underwent a re-evaluation. Intrigued by his dissatisfaction with his patients’ faces, Tweed developed a new diagnostic procedure that changed orthodontics forever, establishing objective criteria to indicate tooth extractions.^5^ Even though nowadays we know that extractions do not guarantee the stability promulgated by Tweed, its need for facial changes or for solving dental crowding was adapted to contemporary orthodontics. Such procedure is sufficiently settled and does not create uproar any longer. Nevertheless, this relative consensus was achieved after much dissatisfaction, disagreement and many doubts.

We have witnessed many other changes, such as those in the 1980’s, with the introduction of prescription brackets and superelastic wires. When these products were launched, to enhance their value in the market, they were called intelligent brackets and wires. At that time, this was interpreted by many professionals as a depreciation of the role of the orthodontist in the treatment. New discussions began, yet the specialty survived and in the end prescription brackets and superelastic wires were adequately assimilated as excellent tools that we choose according to our diagnosis and needs, and in addition to also serving to restructure our clinical practice.

Recently we are living other oscillations, such as the mechanisms for accelerating tooth movement with self-ligating brackets and the idea of reduced friction. 

There are also the vibrating platforms that spur specialists’ hopes in search of faster treatments, even though they are supported by very inconclusive evidences. Still in this line of thought, the general idea that our patients are more like consumers instead of true patients seems to grow,^6^ and that the new digital technologies will replace and annihilate the professional. Maybe, because of the instability that these novelties bring along with them, we are fluctuating between the duality of being doctors or salesmen. But, when treatment planning fails, who enters the scene, the doctor or the salesperson?

In this swirl of trends that encompasses our history, the only certainty that persists is the need for unbalance in search of balance. In this fight between the new that outstands so aggressively and the quietness of what is already settled and seems not to be useful any longer, the result, strange though it may seem, is progress. The dissatisfactions take us to reflections and to changes in direction and, for some time, we stand on what we believe is our point of equilibrium. Until another wave comes and instigates the true need for science.

Good readings!

Flavia Artese - editor-in-chief (flaviaartese@gmail.com)
